# Modulation of Intestinal Histology by Probiotics, Prebiotics and Synbiotics Delivered In Ovo in Distinct Chicken Genotypes

**DOI:** 10.3390/ani11113293

**Published:** 2021-11-18

**Authors:** Monika Bogusławska-Tryk, Ewa Ziółkowska, Anna Sławińska, Maria Siwek, Joanna Bogucka

**Affiliations:** 1 Department of Animal Physiology and Physiotherapy, Faculty of Animal Breeding and Biology, Bydgoszcz University of Science and Technology, 85-084 Bydgoszcz, Poland; Ewa.Ziolkowska@pbs.edu.pl (E.Z.); Joanna.Bogucka@pbs.edu.pl (J.B.); 2 Department of Animal Biotechnology and Genetics, Faculty of Animal Breeding and Biology, Bydgoszcz University of Science and Technology, 85-084 Bydgoszcz, Poland; Anna.Slawinska@pbs.edu.pl (A.S.); Maria.Siwek-Gapinska@pbs.edu.pl (M.S.)

**Keywords:** bioactive substances, in ovo administration, performance indices, intestinal microstructure, broiler chicken, native chicken

## Abstract

**Simple Summary:**

Probiotics, prebiotics and synbiotics are biologically active substances that are commonly used in poultry feeding as an alternative to antibiotic growth promoters. It was found that they could improve the intestinal microstructure as well as the health status and productivity of animals. The aim of this study was to determine the effect of probiotics, prebiotics and synbiotics administrated in ovo on the 12th day of embryonic development on selected morphological parameters of the small intestine in broiler and native chickens. After hatching, the chicks were placed in pens and housed for 42 days. On the last day of the experiment, all birds were individually weighed and slaughtered, and samples for histological analysis were taken from the duodenum, jejunum and ileum. The following parameters were determined: the height, width and surface area of the villi, the thickness of the muscular layer and the depth of the crypts, as well as the ratio of the villi height to the crypt depth. Based on the obtained data, it can be concluded that the substances used have an impact on the production parameters and intestinal morphology in various utility types of poultry. In addition, the obtained results indicate that chickens with different genotypes react differently to a given substance; therefore, the substances should be chosen in relation to the genotype.

**Abstract:**

The aim of the study was to determine the effect of probiotics, prebiotics and synbiotics administered in ovo on selected morphological parameters of the small intestine (duodenum, jejunum, ileum) in broiler chickens (Ross 308) and native chickens (Green-legged Partridge, GP). On the 12th day of embryonic development (the incubation period), an aqueous solution of a suitable bioactive substance was supplied in ovo to the egg’s air cell: probiotic—*Lactococcus lactis* subsp. *cremoris* (PRO), prebiotic—GOS, galacto-oligosaccharides (PRE) or symbiotic—GOS + *Lactococcus lactis* subsp. *cremoris* (SYN). Sterile saline was injected into control (CON) eggs. After hatching, the chicks were placed in pens (8 birds/pen, 4 replicates/group) and housed for 42 days. On the last day of the experiment, all birds were individually weighed and slaughtered. Samples for histological analysis were taken directly after slaughter from three sections of the small intestine. In samples from the duodenum, jejunum and ileum, the height and width of the intestinal villi (VH) were measured and their area (VA) was calculated, the depth of the intestinal crypts (CD) was determined, the thickness of the muscularis was measured and the ratio of the villus height to the crypt depth (V/C) was calculated. On the basis of the obtained data, it can be concluded that the applied substances administered in ovo affect the production parameters and intestinal morphology in broiler chickens and GP. The experiment showed a beneficial effect of in ovo stimulation with a prebiotic on the final body weight of Ross 308 compared to CON, while the effect of the administered substances on the intestinal microstructure is not unequivocal. In GP, the best effect in terms of villi height and V/C ratio was found in the in ovo synbiotic group. Taking into account the obtained results, it can be concluded that chickens of different genotypes react differently to a given substance; therefore, the substances should be adapted to the genotype.

## 1. Introduction

The development, structure and functions of the digestive tract in animals largely depend on the composition and type of diet [1,2,3,4,5,6,7,8,9]. Nutritional factors can both positively and negatively influence the composition of the gut microflora, leading to changes in the end products of the bacterial fermentation of carbohydrates and proteins in the gut [2,3,10,11,12,13,14,15]. The intestinal epithelium, involved in the absorption of nutrients, is also a barrier between the external and internal environment of the organism. Unfavorable changes in the intestinal mucosa, occurring under the influence of the pathogenic bacteria and toxic substances present in the digesta, negatively affect the performance of farm animals [16,17,18].

Pro-, pre- and synbiotics are biologically active substances, which are commonly used as feeding supplements in poultry. Their use increased after the European Union banned antibiotic growth promoters (AGP). Probiotics are preparations that contain live microorganisms, mainly bacteria and yeasts, which, by influencing the composition of the intestinal microbiota, can affect the health of the host [19,20]. Prebiotics are selectively fermented components that positively affect the welfare and health of the host by selectively stimulating the growth and/or activity of the intestinal microflora [12,21,22]. Products containing both probiotics and prebiotics are called synbiotics. Bioactive substances reportedly improve the intestinal microstructure and have a positive effect on the health status and production performance of animals by influencing intestinal microbiota composition and short-chain fatty acids (SCFA) profiles [12,20,21,22,23,24,25], the digestibility of nutrients and the body’s resistance [26,27,28,29,30]. One of the main products of bacterial fermentation, apart from acetic and propionic acid, is butyric acid, which has a beneficial effect on both the digestive tract and peripheral tissues. The activity of butyrate in the organism is related to its regulatory influence on gene expression and limitation of the multiplication of pathogenic bacteria [31,32,33], which may have a beneficial effect on the structure of the intestinal mucosa. Studies conducted in recent years indicated the antibacterial, immunostimulatory and antidiarrheal effects of bioactive substances [34,35,36,37]. Such a pro-health effect, also found in poultry, results from their beneficial effects on the microflora of the gastrointestinal tract and the microstructure of the intestinal mucosa [38,39,40]. 

According to de Vrese and Schrezenmeir [41] and Yang et al. [42], the effectiveness of pro-, pre- and synbiotics operation depends both on the composition of the preparation as well as the time and method of their administration. In poultry production, biologically active substances are usually added to feed and water. The effectiveness of this type of delivery was documented in numerous studies. For example, it was found that the probiotics, prebiotics and synbiotics administered in-feed had a positive effect on the development and broiler performance, mediated by stimulated intestinal microflora, immune system and small intestine mucosa [43,44,45,46,47]. An alternative to the oral administration of bioactive substances is the in ovo method. Numerous studies show that the delivery of pro, pre- and/or synbiotics to the egg during the embryonic development of the chick has a positive effect on the development of the digestive tract [7] and the immune status of birds [48,49,50,51,52,53]. The in ovo-delivered bioactive substances primarily influence the composition of the intestinal microflora [54,55], but their effect depends on the time point of delivery and the composition of the injected compounds/probiotic strains [56].

As a result of intensive breeding work, two utility lines were obtained: high-laying chickens (laying hens) and chickens of the meat type (broilers). The growth rates of the two production types were different, presumably due to the characteristic development of the digestive system. The studies by Uni et al. [57] showed that the intestinal morphological parameters, such as the height and width of the intestinal villi, as well as the depth of the crypts and the size of the absorption area, could be related to the greater body weight of broilers compared to laying hens. Additionally, Simon et al. [58] showed that the genetic background could influence the ileal IgA, IgM and IgY expressions, which were higher in broiler chickens compared to laying hens. These differences were most likely attributed to the differences in the gut microbiota composition between distinct chicken genotypes [59]. There are few reports in the available literature comparing the physiological parameters of commercial chickens with native breeds. It is known, however, that native breeds are characterized by a greater resistance and better ability to adapt to environmental conditions [60]. The chicken breed native to Poland is the Green-legged Partridge (GP), which is treated as general-purpose poultry, characterized by a high tolerance to very low temperatures and extensive rearing. GP are phenotypically and genetically distinct compared to highly selected broiler chickens [61]. 

In this study, we hypothesized that there is a relation between the bioactive substances administered in ovo and chicken genotype expressed in the microstructure of the small intestine in broiler chickens and native chickens. This study aimed to determine the effect of probiotics, prebiotics and synbiotics administered in ovo on selected morphological parameters of the small intestine in broiler chickens and GP chickens.

## 2. Materials and Methods

### 2.1. Animal Procedures

The research was carried out on broiler chickens (Ross 308) and native chickens (Green-legged Partridge, GP). The experiment began with egg incubation (600 eggs/genotype) in a commercial hatchery using an automated incubator at 37.8 °C and a relative humidity of 61–63%. The broiler eggs were obtained from a commercial breeding flock, while the GP eggs came from the conservation flock managed by the University of Life Sciences in Lublin, Poland. On day 12 of egg incubation, aquatic solutions of the respective bioactive substance was delivered in ovo into the eggs’ air cells: probiotic (*Lactococcus lactis* subsp. *cremoris*, 10^5^ CFU/egg), prebiotic (GOS, galactooligosaccharides, 3.5 mg/egg), or synbiotic (GOS, 3.5 mg /egg + *Lactococcus lactis* subsp. *cremoris*, 10^5^ CFU/egg). The selection of the bioactive compounds and their doses was based on previous research [55,62]. GOS was derived from Clasado Biosciences Ltd. (Jersey, UK) and is known under trade name: Bi^2^tos. *Lactococcus lactis* subsp. *cremoris*. IBB477 was obtained from the collection of the Institute of Biochemistry and Biophysics Polish Academy of Sciences (Warsaw, Poland). Sterile physiological saline was injected into the control eggs. The injection volume was 0.2 mL for each egg. After in ovo injection, the puncture hole was sealed, and incubation continued. Detailed information on the in ovo procedure is presented in Sławińska et al. [63]. 

The experiments were conducted at an experimental farm of Wroclaw University of Environmental and Life Sciences (Wroclaw, Poland) with the consent of the Local Ethics Committee for Animal Experiments (Bydgoszcz, Poland, no. 16/2014). After hatching, the chicks were placed in deep litter pens with a surface area of 3.75 m^2^, and with a stocking rate of 17.33 birds/m^2^ (32 birds per group: 8 birds/pen, 4 replicates/group) where they were kept for 42 days. All pens had the same environmental conditions. During the course of the experiment, both Ross 308 and GP were fed standard diets (Table 1), and both feed and fresh water were available ad libitum. The environmental conditions were adjusted to the age of the birds. At the end of the experiment (42 d of age), all birds were individually weighed, stunned and slaughtered. Samples for histological analysis (ca. 3–4 cm) were taken immediately after slaughter from three segments of the small intestine: from the midpoint of the duodenum, the midpoint between the point of entry of the bile duct and Meckel’s diverticulum (jejunum), and midway between Meckel’s diverticulum and the ileocecal junction (ileum).

### 2.2. Analytical Methods

Individual segments of the intestine were rinsed with 0.9% physiological saline, and then fixed with 4% CaCO_3_ buffered formalin for 24 h. Then, they were dehydrated in graded ethanol series, cleaned in xylene and infiltrated with paraffin in a tissue processor (Thermo Shandon, Chadwick Road, Astmoor, Runcorn, Cheshire, UK), and then embedded in paraffin wax (Medite, Burgdorf, Germany). Samples were cut on scraps at 10-μm thickness using a microtome (Thermo Shandon, Chadwick Road, Astmoor, Runcorn, Cheshire, UK) and mounted on microscope slides coated with an egg albumin and glycerin mixture. Sections were stained with periodic acid-Schiff (PAS) for morphometric evaluation.

The measurements of the height and width of the villi, crypt depth and muscle thickness were performed using a Nikon Ci-L microscope equipped with a Nikon DS-Fi3 camera with a resolution of 5.9 MPix and NIS ELEMENTS software. Next, the villi area (VA) was calculated using the formula cited by Sakamoto et al. [64]:

VA = 2π × (VW/2) × VH,

where VW = villus width, and VH = villus height. The villus height/crypt depth ratio (V/C) was also calculated. Linear measurements of the thickness of the muscular layer of small intestine were conducted on five consecutive slices.

### 2.3. Statistical Analysis

The data are presented as means and standard deviation (SD). The results were statistically analyzed by one-way ANOVA using STATISTICA 13.1 software (StatSoft^®^, Tulsa, OK, USA). For data that corresponded with the normal distribution, the post hoc Dunkan’s multiple range test was applied. All differences were considered significant at *p* < 0.05.

## 3. Results

### 3.1. Performance Indices of Ross 308 and GP Chickens Stimulated In Ovo with Pro-, Pre- and Synbiotics

Table 2 presents the performance indices of 42-day-old Ross 308 and GP stimulated in ovo with pro-, pre- and synbiotics. In the conducted experiment, a significant effect of in ovo stimulation with prebiotics and synbiotics on the final body weight of Ross 308 was found. In PRE, the body weight of Ross 308 was the highest (*p* < 0.05), and in SYN it was the lowest (*p* < 0.05), compared to CON. Similar relationships were found in GP, but without the significant effects of in ovo stimulation on the final body weight (*p* > 0.05). There was also no effect of the tested factor on feed intake (FI) and feed conversion rate (FCR) in both Ross 308 and GP chickens.

### 3.2. Histological Parameters of Small Intestine of Ross 308 and GP Chickens Stimulated In Ovo with Pro-, Pre- and Synbiotics

The histological parameters of three segments of the small intestine (duodenum, jejunum and ileum) in 42-day-old Ross 308 and GP chickens are presented in Table 3 and Table 4. In the conduced experiment, the effects of the additives used in ovo on the villus height and width, villus area, crypt depth, muscle thickness and V/C ratio in the duodenum and jejunum of Ross 308, and in the duodenum of GP chickens, were not determined (*p* > 0.05). There were significant effects of the stimulation in ovo with pro-, pre- and synbiotics on the morphometric parameters in the ileum of Ross 308 and in jejunum and ileum of GP chickens (*p* < 0.05). 

### 3.3. Effect of Chickens Genotype on Histological Parameters of Small Intestine

**Figure 1:**
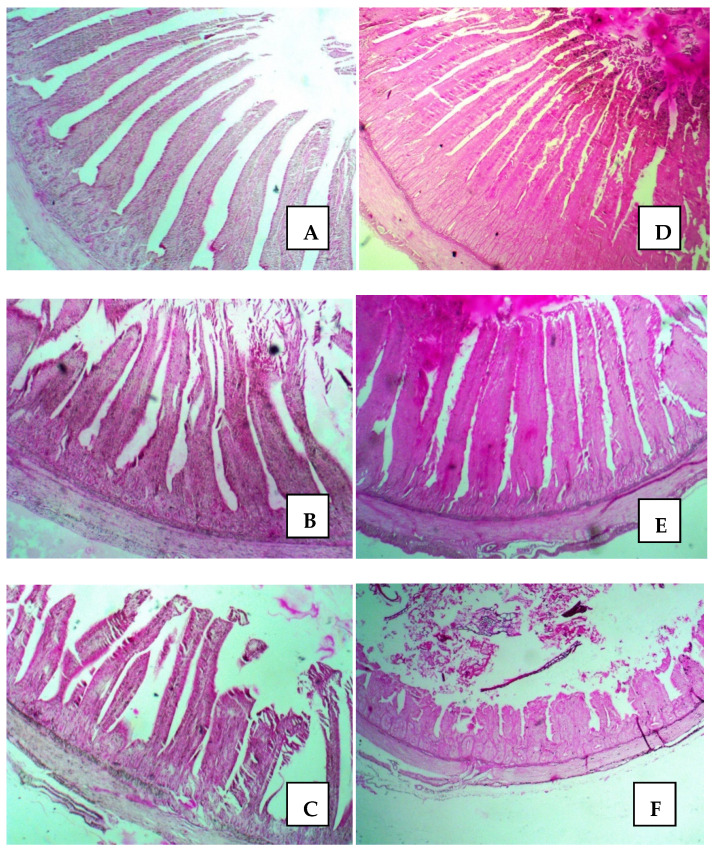
Microstructure of small intestine of Ross 308 chickens (**A**) duodenum, (**B**) jejunum, (**C**) ileum and GP chickens (**D**) duodenum, (**E**) jejunum, (**F**) ileum; PAS stain; magnification ×40.

By analyzing the influence of the genotype on the histological parameters of the small intestine (Table 5), we found that the height of the villi, the area of the villi and the muscle thickness were greater in Ross 308 than in GP chickens (*p* < 0.05), regardless of the group (Figure 1). The intestinal villi width was greater in the duodenum in Ross 308 from the CON and PRO groups, and in the jejunum from PRE and SYN groups compared to GP chickens (*p* < 0.05). The V/C ratio was more favorable in the duodenum of Ross 308 from CON, PRE and SYN groups, and in jejunum of Ross 308 from CON and PRE, compared to the GP (*p* < 0.05). In the ileum, apart from the control group, no evidence was found for the influence of the genotype on V/C ratio in the experimental birds (*p* > 0.05).

## 4. Discussion

We conducted a histological study in two distinct genotypes of chickens stimulated in ovo with prebiotics, probiotics and synbiotics. The obtained results clearly indicated that the development of the intestinal morphology depended on the substance applied in ovo as well as on the chicken genotype. 

### 4.1. Performance Indices of Ross 308 and GP Chickens Stimulated In Ovo with Pro-, Pre- and Synbiotics

In the conducted study, the in ovo stimulation with prebiotics significantly increased the FBW of 42-day-old Ross 308, compared to CON and SYN groups. These results correlated with the results of studies by Bogucka et al. [7] conducted on broiler chickens injected in ovo with bioactive substances. The authors found a beneficial effect of in ovo administered transgalacto-oligosaccharides on the final body weight of 35-day-old Ross 308 chickens compared to the control, inulin and synbiotic groups. On the other hand, Maiorano et al. [65] and Berrocoso et al. [66] did not find effects of raffinose family oligosaccharides (RFO) administered in ovo on the BW of 42-day-old Ross 308 and 21-day-old Cobb 500. Additionally, Miśta et al. [67] found that the in ovo injections of prebiotics did not affect broiler body weight. Other studies carried out on meat-type chickens confirmed the beneficial effect of adding prebiotics to feed on the performance indicators of broilers. Nabizadeh [14] showed that the inclusion of 1% inulin, but not 0.5%, into the diet of broilers significantly increased body weight after 42 days of rearing. Rebole et al. [68] demonstrated that a diet supplemented with inulin (at a level of 1%) positively affected the BWG of broilers. Moreover, Mookiah et al. [46] noted improvements in body weight gains in broiler chickens that were fed a diet supplemented with isomalto-oligosaccharides, compared to control broiler chickens. Xu et al. [16] observed improvements in the body weight gain of broiler chickens fed a diet with 4% of fructooligosaccharides (FOS). The higher body weight obtained in the experiment in chickens stimulated in ovo with the prebiotics can be explained by its beneficial effect on the organism. Numerous studies show that the positive effect of prebiotic substances administered in ovo or in feed is associated with the growth of beneficial bacteria, as well as a reduction in the number of potential pathogens in the intestines and the improvement of immune functions. Substances with prebiotic effects also affect the condition and functions of the digestive tract by increasing the secretion of digestive enzymes [69], thus affecting the digestibility and absorption of nutrients. In addition, volatile fatty acids, resulting from the fermentation of prebiotics, have a positive effect on the structure and functions of the small intestines [7,66,70]. 

### 4.2. Histological Parameters of Small Intestine of Ross 308 and GP Chickens Stimulated In Ovo with Pro-, Pre- and Synbiotic

The small intestine is a barrier that separates the internal environment of the body from the external environment. It is also an organ highly specialized in the digestion and absorption of nutrients. In poultry, the small intestine is relatively short, and the absorption area mainly depends on the surface area of the intestinal villi [71]. The structure of the small intestine provides important information on the health status of the digestive tract. Due to the proximity of the mucosa surface and the intestinal contents, dietary toxic substances and pathogenic bacteria may affect the condition of the intestinal mucosa, and their effect may be manifested in changes of the structure of the intestinal villi and the depth of the crypts [16]. 

In this study, a statistically significant increase in villi width in the jejunum in GP after the in ovo administration of probiotics resulted in the largest intestinal villi area. This may indicate a beneficial effect of probiotics on the absorption surface in the jejunum of GP chickens. It is well known that probiotics and other bioactive substances improve the morphological parameters of the intestinal mucosa and may have a beneficial effect on the absorption surface. This may be due to the competition for resources and ecological niche between lactic acid bacteria (LAB) and pathogenic microorganisms. This competition is mainly based on the ability of LAB to produce volatile fatty acids and bacteriocins that inhibit the growth of pathogenic bacteria [72]. Yang et al. [24] found a linear relationship between the amount of *Bacillus* and *Salmonella* bacteria in the cecum of chickens. Awad et al. [27] indicated that probiotics had beneficial effects on the morphology of the intestine and protected against pathogenic bacteria, but also improved the electrophysiology of the small intestine. 

The effect of the bioactive substances administered in ovo in the ileum of GP chickens was not clear and difficult to explain. The elongation of the intestinal villi in the SYN group did not increase their surface area. This was likely due to the fact that the in ovo administration of synbiotic did not influence the width of the villi in the third segment of the small intestine. The largest villi area was recorded in native chickens injected with prebiotics, which were also characterized by the highest body weight. Prebiotics are selectively fermentable substances that modify the composition and activities of the gastrointestinal microbiota [21]. The most common prebiotics include inulin, galacto-, fructo- and mannan-oligosaccharides, as well as raffinose-oligosaccharides [12,22,55]. These substances are fermented by commensal bacteria, and the volatile fatty acids produced in this process primarily decrease the pH of the intestinal contents [73]. One of the SCFA produced by the microbiota is the butyrate, which stimulates the growth of intestinal epithelial cells, and thus improves nutrient absorption [74]. 

The ratio of the crypt depths to the villi height is an indicator of the digestive potential of the gut and may indicate the maturity of the intestinal mucosa [1]; the elongation of the villi increases the area of nutrient absorption [26]. Additionally, shallower crypts may indicate a lower loss of enterocytes from the villi surface, which slows down the mitosis of cryptographic cells. In studies conducted on broiler chickens with the use of bioactive substances, Awad et al. [26,27] found that their use in bird nutrition significantly elongated the villi and increased the V/C ratio in the ileum and duodenum compared to the control group. According to the authors, this is of practical and economic importance, because shortening the intestinal villi with the simultaneous deepening of the crypts may lead to a reduction in the absorption of nutrients and in the efficiency of meat chickens. In the experiment there was no beneficial effect of bioactive substances administered in ovo at the V/C ratio in Ross 308 chickens. Therefore, when analyzing the effect of in ovo stimulation with various bioactive substances in Ross 308, it is difficult to explain a significant decrease in the height, width and surface of the villi, as well as the V/C ratio in the ileum in the group stimulated with synbiotics. The most favorable effect of the in ovo application of the bioactive substance on the V/C ratio in the jejunum and ileum of GP chickens was noted in the SYN group; although, in the case of pro- and prebiotics, their positive effect on the intestinal microstructure was also observed. This may prove the beneficial effect of the preparations on the structure of the small intestine of GP chickens. So far, most of the experiments looking at the effect of in ovo stimulation with various bioactive substances have been carried out on broiler chickens [50,53,56,65,75]. However, few studies indicated that probiotics may also positively affect the health of the small intestine mucosa in laying hens [76,77,78]. The studies of Lei et al. [77] and Xiang et al. [78] found, for example, that feeding laying hens a diet supplemented with *Clostridium butyricum* and a combination of *Saccharomyces boulardii*, *Pediococcus acidilactici* and *B. licheniformis* resulted in a favorable ratio of villi height to the depth of the crypts in the ileum and cecum. 

### 4.3. Effect of Chickens Genotype on Histological Parameters of Small Intestine

The morphometric and morphological parameters of the gastrointestinal tract, in particular the small intestine, may be significantly influenced by the composition of the diet and the supplements used, including pro-, pre- and synbiotics [7,38,79]. Moreover, the genotype of birds is a factor determining the differences in production and physiological parameters in poultry [48]. It was shown that the deposition of pectoral muscles and their chemical composition differed depending on the genotype and sex of the bird [80]. It is well known that laying-type, dual-purpose and meat-type chickens differ significantly in terms of physiological and production parameters. This is due to the intensive selection of birds in terms of the production of eggs or meat. This results in large differences in the growth rates of the various types of poultry. The average body weight of a 6-week-old broiler chicken is 2918 g [81], while the laying-type of chicken weighs, on average, 432 g [82]. In addition, the selection of birds for specific performance traits may affect the structure and functions of the digestive tract and the function of the immune system [57]. This study showed significant differences between two distinct chicken genotypes (Ross 308 vs. GP) stimulated in ovo. The conditions in which the chickens were kept during the experiment were identical for both genotypes. It is known that Ross 308 are characterized by an intensive growth rate and high production parameters. However, the long hatching window and the lack of access to food and water may disrupt the development of the intestinal microflora due to the limited possibility of microbiota inoculation [83]. On the other hand, GP is a general-purpose chicken breed, with low nutritional and environmental requirements, characterized by a much slower growth rate and better disease resistance compared to broiler chickens [61]. These two types of chickens differ significantly in the histological parameters of the small intestine, which is likely due to their genetic background. The longer villi and the deeper crypts in Ross 308 compared to GP chickens correspond with the results obtained by Uni et al. [57]. These authors found more favorable intestinal morphological parameters in meat-type chickens compared to laying-type chickens. The differences in villi length and crypt depth between Ross 308 and GP may be a consequence of the origin of the birds. Broiler chickens and laying hens were created through intensive selection and are adapted to farm rearing, while GP, as an old, native breed, is mainly used for extensive rearing because it forages well and is resistant to disease. This form of maintenance and feeding with fodder of a lower nutritional value higher fiber content than farm fodder may result in the less intensive development of the intestinal microstructure.

## 5. Conclusions

Based on the data obtained, it can be concluded that pre-, pro- and synbiotics administered in ovo have an impact on the production parameters and intestinal morphology in Ross 308 and GP chickens. However, it is difficult to determine which of the compounds used has the best effect on the microstructure of the small intestine. In Ross 308, the effect of pro, pre- and synbiotics is not unequivocal, while in GP chickens the largest effect, taking into account the height of the villi and the V/C ratio, was determined in the groups administered in ovo either with prebiotic or synbiotic. Taking into account the obtained results, it can be concluded that chickens with different genotypes react differently to pro-, pre- and synbiotics; therefore, the substances should be adapted to the genotype/breed.

## Figures and Tables

**Table 1:** Analyzed chemical composition of the basal diet (%) used in Ross 308 and GP chickens feeding from the 1st to the 42nd day of rearing.

Day of Rearing	Analyzed Chemical Composition (%)			
	CP	CF	LYS	MET
Ross 308				
1−10	21.20	3.04	1.25	0.56
11−34	19.51	3.04	1.16	0.52
35–42	18.33	3.24	1.05	0.47
GP				
1–28	19.50	3.40	1.09	0.44
28–42	18.99	3.50	0.99	0.43

CP—crude protein; CF—crude fiber; LYS—L-Lysine; MET—DL-Methionine.

**Table 2:** Performance indices of Ross 308 and GP chickens stimulated in ovo with pro-, pre- and synbiotics.

Group	Performance Indices (Mean ± SD)			
	FBW, g	FI, g	FCR	Mortality %
Ross 308				
CON	3129.4 ^b^ ± 219.6	4673.2 ± 118.1	1.50 ± 0.07	4.7
PRO	3229.7 ^ab^ ± 320.7	4832.7 ± 198.0	1.49 ± 0.04	3.1
PRE	3277.1 ^a^ ± 325.2	5080.5 ± 131.7	1.55 ± 0.03	7.7
SYN	2978.5 ^c^ ± 243.7	4949.7 ± 231.0	1.66 ± 0.05	4.6
GP				
CON	446.2 ± 77.7	940.7 ± 74.2	2.1 ± 0.1	6.3
PRO	448.8 ± 47.4	937.5 ± 41.7	2.1 ± 0.2	0.0
PRE	465.3 ± 57.1	1059.7 ± 131.7	2.3 ± 0.3	9.4
SYN	419.2 ± 56.2	1106.0 ± 90.9	2.5 ± 0.3	9.4

SD—standard deviation; FBW—final body weight, 42 d of rearing; FI—feed intake; FCR—feed conversion rate; CON—control; PRO—probiotic (*L. lactis* subsp. *cremoris*); PRE—prebiotic (GOS, galactooligosaccharides); SYN—synbiotic (GOS+ *L. lactis* subsp. *cremoris*); ^a^, ^b^, ^c^—mean values in the columns marked with different letters differ significantly (*p* < 0.05).

**Table 3:** Histological parameters of small intestine of Ross 308 chickens stimulated in ovo with pro-, pre- and synbiotics.

Item	Duodenum	Jejunum	Ileum
	Mean ± SD		
Villus height (µm)			
CON	1976.0 ± 175.3	1623.7 ± 140.7	1306.4 ± 220.2 ^a^
PRO	2054.1 ± 197.2	1624.5 ± 137.7	1150.4 ± 132.3 ^ab^
PRE	2015.4 ± 169.2	1640.0 ± 171.8	1204.8 ± 95.4 ^ab^
SYN	1968.9 ± 245.4	1594.1 ± 179.4	1007.9 ± 95.5 ^bc^
Villus width (µm)			
CON	223.5 ± 17.0	199.8 ± 27.3	168.7 ± 16.3 ^a^
PRO	228.6 ± 19.0	203.5 ± 26.6	177.5 ± 15.3 ^a^
PRE	214.7 ± 27.5	178.6 ± 21.7	174.3 ± 17.6 ^a^
SYN	208.6 ± 23.8	179.2 ± 17.2	145.7 ± 10.9 ^b^
Villus area (µm^2^)			
CON	1,384,693 ± 158,348.4	1,020,554 ± 161,838.1	696,609.0 ± 159,256.6 ^a^
PRO	1,475,097 ± 164,759.4	1,030,014 ± 99,849.3	637,960.0 ± 58,999.5 ^a^
PRE	1,360,305 ± 230,192.6	920,610 ± 157,747.8	662,883.4 ± 102,808.2 ^a^
SYN	1,281,808 ± 170,431.5	901,080 ± 162,607.3	459,453.5 ± 37,217.9 ^b^
Crypt depth (µm)			
CON	180.6 ± 17.7	150.5 ± 8.4	149.3 ± 22.7
PRO	158.4 ± 18.0	160.2 ± 18.3	134.7 ± 13.8
PRE	167.7 ± 16.5	150.9 ± 16.8	132.8 ± 15.7
SYN	159.5 ± 20.4	154.7 ± 12.2	131.1 ± 9.5
Muscle thickness (µm)			
CON	222.9 ± 64.9	200.0 ± 34.8	197.5 ± 37.2
PRO	223.3 ± 63.5	173.2 ± 29.2	178.9 ± 26.3
PRE	272.8 ± 58.1	202.1 ± 29.9	197.3 ± 45.4
SYN	294.0 ± 63.5	206.3 ± 34.7	213.3 ± 36.6
V/C			
CON	11.2 ± 1.5	10.6 ± 1.1	8.8 ± 0.9 ^a^
PRO	13.1 ± 1.2	10.4 ± 1.2	8.5 ± 0.9 ^a^
PRE	12.1 ± 1.6	10.9 ± 1.7	9.1 ± 0.6 ^a^
SYN	12.4 ± 1.5	10.3 ± 0.9	7.7 ± 1.1 ^b^

SD—standard deviation; CON—control; PRO—probiotic (*L. lactis* subsp. *cremoris*); PRE—prebiotic (GOS, galactooligosaccharides); SYN—synbiotic (GOS + *L. lactis* subsp. *cremoris*); V/C—villus height/crypt depth ratio; ^a^, ^b^, ^c^—mean values in the columns marked with different letters differ significantly (*p* < 0.05).

**Table 4:** Histological parameters of small intestine of GP chickens stimulated in ovo with pro-, pre- and synbiotics.

Item	Duodenum	Jejunum	Ileum
	Mean ± SD		
Villus height (µm)			
CON	1565.7 ± 128.3	867.9 ± 180.7	397.1 ± 59.4 ^c^
PRO	1556.4 ± 258.4	1051.2 ± 224.6	568.0 ± 263.9 ^bc^
PRE	1415.7 ± 273.7	919.3 ± 264.7	766.4 ± 298.7 ^ab^
SYN	1411.3 ± 202.9	1135.1 ± 222.7	836.2 ± 162.6 ^ab^
Villus width (µm)			
CON	174.5 ± 19.9	165.1 ± 37.2 ^ab^	172.0 ± 31.7
PRO	179.4 ± 18.5	206.6 ± 42.8 ^a^	172.6 ± 20.4
PRE	189.6 ± 22.6	146.4 ± 34.2 ^b^	165.6 ± 45.3
SYN	188.7 ± 29.4	145.2 ± 29.3 ^b^	133.6 ± 37.5
Villus area (µm^2^)			
CON	860,859.2 ± 138,231.1	451,569.9 ± 144,440.1 ^b^	215,643.7 ± 55,405.5 ^b^
PRO	888,202.4 ± 247,938.4	665,555.0 ± 120,680.5 ^a^	305,713.0 ± 131,553.9 ^b^
PRE	836,758.5 ± 170,742.5	433,556.3 ± 187,185.2 ^b^	416,139.1 ± 228,113.1 ^a^
SYN	843,224.0 ± 226,409.5	516,589.0 ± 129,337.3 ^ab^	335,962.0 ± 42,864.8 ^ab^
Crypt depth (µm)			
CON	143.6 ± 17.1	106.5 ± 11.5	76.7 ± 12.7
PRO	136.6 ± 23.0	109.4 ± 30.1	78.7 ± 17.5
PRE	146.3 ± 19.7	106.0 ± 15.7	95.0 ± 18.7
SYN	138.3 ± 15.7	109.4 ± 9.8	90.1 ± 6.1
Muscle thickness (µm)			
CON	152.7 ± 51.9	137.1 ± 45.7	106.2 ± 32.0
PRO	142.4 ± 33.4	120.4 ± 17.3	102.7 ± 29.2
PRE	153.8 ± 27.2	110.9 ± 23.7	114.4 ± 13.5
SYN	153.2 ± 30.7	133.8 ± 14.7	128.3 ± 7.8
V/C			
CON	9.0 ± 1.0	8.1 ± 1.6	5.3 ± 1.1 ^c^
PRO	8.6 ± 0.8	9.9 ± 2.7	7.0 ± 1.9 ^bc^
PRE	7.6 ± 1.9	8.3 ± 2.2	8.0 ± 2.8 ^ab^
SYN	7.5 ± 0.9	10.4 ± 1.9	9.3 ± 1.8 ^ab^

SD—standard deviation; CON—control; PRO—probiotic (*L. lactis* subsp. *cremoris*); PRE—prebiotic (GOS, galactooligosaccharides); SYN—synbiotic (GOS + *L. lactis* subsp. *cremoris*); V/C—villus height/crypt depth ratio; ^a^, ^b^, ^c^—mean values in the columns marked with different letters differ significantly (*p* < 0.05).

**Table 5:** Effect of chicken genotype on histological parameters of small intestine.

Item	Genotype ^1^ *p* Value ^2^		
	Duodenum	Jejunum	Ileum
Villus height (µm)			
CON	0.0001	0.00001	0.00001
PRO	0.0014	0.00001	0.00001
PRE	0.0001	0.00001	0.0031
SYN	0.0003	0.0001	0.0180
Villus width (µm)			
CON	0.00001	NS	NS
PRO	0.0002	NS	NS
PRE	NS	0.0382	NS
SYN	NS	0.0188	NS
Villus area (µm^2^)			
CON	0.00001	0.00001	0.00001
PRO	0.0002	0.00001	0.00001
PRE	0.0001	0.00001	0.0183
SYN	0.0011	0.00001	0.0001
Crypt depth (µm)			
CON	0.0003	0.00001	0.00001
PRO	NS	0.0019	0.00001
PRE	0.0336	0.00001	0.0015
SYN	0.0414	0.00001	0.00001
Muscle thickness (µm)			
CON	0.0409	0.0078	0.0001
PRO	0.0041	0.0015	0.00001
PRE	0.0001	0.00001	0.0003
SYN	0.00001	0.0001	0.00001
V/C			
CON	0.0397	0.0016	0.00001
PRO	NS	NS	NS
PRE	0.0266	0.0226	NS
SYN	0.0047	NS	NS

^1^ Genotype: Ross 308 vs. GP chickens; ^2^ For significantly different data, the *p*-value was given, significance level: *p* < 0.05 and *p* > 0.05 (non-significant, NS). The significance of effects was calculated with one-way ANOVA. CON—control; PRO—probiotics (*L. lactis* subsp. *cremoris*); PRE—prebiotics (GOS, galactooligosaccharides); SYN—synbiotics (GOS + *L. lactis* subsp. *cremoris*); V/C—villus height/crypt depth ratio.

## Data Availability

The data presented in this study are available on request from the corresponding author.
